# Molecular Typing and Virulence Characteristic of Methicillin-Resistant *Staphylococcus Aureus* Isolates from Pediatric Patients in Bucaramanga, Colombia

**DOI:** 10.1371/journal.pone.0073434

**Published:** 2013-08-23

**Authors:** Mayra Alejandra Machuca, Luis Miguel Sosa, Clara Isabel González

**Affiliations:** 1 Grupo de Inmunología y Epidemiología Molecular, GIEM, Facultad de Salud, Universidad Industrial de Santander, Bucaramanga, Colombia; 2 Grupo PAIDOS, Departamento de Pediatría, Hospital Universitario de Santander, Facultad de Salud, Universidad Industrial de Santander, Bucaramanga, Colombia; Fundacion Huesped, Argentina

## Abstract

**Background:**

*Staphylococcus aureus* is among the most common global nosocomial pathogens. The emergence and spread of methicillin-resistant *Staphylococcus aureus* (MRSA) is a public health problem worldwide that causes nosocomial and community infections. The goals of this study were to establish the clonal complexes (CC) of the isolates of MRSA obtained from pediatric patients in a university hospital in Colombia and to investigate its molecular characteristics based on the virulence genes and the genes of staphylococcal toxins and adhesins.

**Methods:**

A total of 53 MRSA isolates from pediatric patients with local or systemic infections were collected. The MRSA isolates were typed based on the SCCmec, MLST, *spa* and *agr* genes. The molecular characterization included the detection of Panton-Valentine Leukocidin, superantigenic and exfoliative toxins, and adhesin genes. The correlation between the molecular types identified and the profile of virulence factors was determined for all isolates.

**Results:**

Four CC were identified, including CC8, CC5, CC80 and CC78. The ST8-MRSA-IVc-*agr*I was the predominant clone among the isolates, followed by the ST5-MRSA-I-*agr*II and ST5-MRSA-IVc-*agr*II clones. Twelve *spa* types were identified, of which t10796 and t10799 were new repeat sequences. The isolates were carriers of toxin genes, and *hlg* (100%), *sek* (92%) and *pvl* (88%) were the most frequent. Ten toxin gene profiles were observed, and the most frequent were *seq-sek-hlg* (22.6%), *sek-hlg* (22.6%), *seb-seq-sek-hlg* (18.9%) and *seb-sek-hlg* (15.1%). The adhesion genes were present in most of the MRSA isolates, including the following: *clf-A* (89%), *clf-B* (87%), *fnb-A* (83%) and *ica* (83%). The majority of the strains carried SCCmec-IVc and were identified as causing nosocomial infection. No significant association between a molecular type and the virulence factors was found.

**Conclusion:**

Four major MRSA clone complexes were identified among the isolates. ST8-MRSA-IVc-*agr*I *pvl+* (USA300-LV) was the most frequent, confirming the presence of community-associated MRSA in Colombian hospitals.

## Introduction

*Staphylococcus aureus* is an important cause of a wide variety of diseases, including skin and soft-tissue infections, osteomyelitis, necrotizing pneumonia, bacteremia and endocarditis [1]. The clinical importance of *S. aureus* is attributed to its high virulence and its rapid development of drug resistance. Surface proteins, toxins, and enzymes [2] are virulence factors involved in the infection process.

Since methicillin-resistance *S. aureus* (MRSA) was identified for the first time in 1961, it has become the most common cause of nosocomial and community infection worldwide [3]. Patients with invasive MRSA infections have a higher mortality rate than those with non-invasive MRSA infection or methicillin-sensible *S. aureus* (MSSA) infection [4].

A number of molecular methods have been developed and implemented to type MRSA isolates, including staphylococcal cassette chromosome mecA typing (SCCmec) [5], pulsed-field gel electrophoresis (PFGE) [6], multilocus sequence typing (MLST) [7], *spa* typing and identification of the accessory gene regulator (*agr*) [8]. These typing methods have been used to identify the major clones disseminated globally and to monitor the evolutionary process of these pandemic clones. Some of the pandemic clones identified worldwide include the New York/Japan (ST5-MRSA-II), Hungarian (ST239-MRSA-III), Pediatric (ST5-MRSA-IV) and USA 300 (ST8-MRSA-IVa) clones [9], [10]; in Latin America, the Brazilian (ST239-MRSA-III) and Cordobes/Chileno (ST5-MRSA-IV) clones predominate [11].

MRSA is a growing health problem in Colombian hospitals, in which its prevalence is approximately 40–60% [12], [13], [14]; few molecular epidemiology analyses have been performed. Previous studies have focused on MRSA typing using PFGE and SCCmec and have selected a limited number of representative isolates to be analyzed by MLST [15], [16]. Several MRSA clones associated with nosocomial infection have been described, including the Pediatric, Brazilian, Chilean and USA-300 clones [15]–[17]. The USA-300 clone detected in Latin American is a variant (USA-300 LV) that carries the SCCmec IVc; exhibits high resistance to tetracycline and an absence of the arginine catabolic mobile element (ACME); and has been reported as a predominant clone in the 5 major cities of Colombia [17], [18]. The Pediatric clone has been detected in a significant percentage of cases, and it has a multi-resistant phenotype with resistance to several non-beta lactam antibiotics such as ciprofloxacin, clindamycin, erythromycin, gentamicin, rifampicin, tetracycline, chloramphenicol and trimethoprim/sulfamethoxazole [16].

MRSA expresses different virulence factors involved in the pathogenesis of *S. aureus* infection. These virulence factors are associated with attachment, persistence, the evasion/destruction of host defenses, tissue invasion/penetration and toxin-mediated disease [19]. The studies mentioned above and others described in Colombia have performed MRSA typing to identify the circulating clonal complex in hospitals, but correlation of the genotype with the phenotype and virulence factors has not been performed. We performed molecular typing using MLST, the *spa* gene, SCCmec and *agr* in a cohort of MRSA isolates obtained from pediatric patients hospitalized in a university hospital from Bucaramanga, Colombia and correlated the molecular clones identified with their phenotypes and virulence factors.

## Materials and Methods

### Clinical isolates and antimicrobial susceptibility test

A total of 53 MRSA isolates were collected from pediatric patients with local or systemic infections in a university hospital in Bucaramanga, Colombia from March 2007 to March 2009. All of the isolates were individual cases that were epidemiologically unrelated. MRSA was identified by an automated method (BD PhoenixTM 100, USA) and confirmed by polymerase chain reaction (PCR) to harbor the *nuc*A and *mec*A genes. The antimicrobial susceptibility pattern was determined by the disc diffusion test using the following antibiotics: clindamycin, ciprofloxacin, gentamicin, amikacin, tetracycline, rifampicin and trimethoprim/sulfamethoxazole, in accordance with the guidelines of the Clinical and Laboratory Standards Institute (CLSI) [20]. Inducible macrolide-lincosamide-streptogramin B resistance (MLSBi) was determined by the D-test, and the vancomycin MICS were evaluated. Once the isolates were confirmed, the infection type was classified using clinical and epidemiologic information obtained from medical records. The infection type was classified as community-associated (CA), healthcare-associated infection community onset (HACO) or hospital onset (HAHO), according to standard epidemiological definitions established by the U.S. Centers for Disease Control and Prevention (CDC) [21]. The infections were classified according to the following definitions:

CA-MRSA: Clinical condition and isolation of MRSA without healthcare-associated risk factors.
HACO-MRSA: Isolation of MRSA within the first 48 hours of hospital admission and at least one of the following health care risk factors: the presence of an invasive device at the time of admission, a history of MRSA infection, surgery, hospitalization, dialysis, or intensive care unit (ICU) admission during the 12 months preceding the culture date.
HAHO-MRSA: Isolation of MRSA after 48 hours of hospital admission and at least one of the risk factors mentioned above [21].

### DNA isolation and SCC*mec* typing

Genomic DNA was extracted from each MRSA isolate and grown in BHI broth overnight at 37°C. The bacterial pellet was suspended in 0.5 mM EDTA (pH 8) and digested with lysostaphin (2 mg/ml), lysozyme (20 mg/ml) and proteinase K (20 mg/ml). DNA was extracted and purified by alcohol precipitation with 2-propanol and ethanol. The DNA was quantified by 260/280 spectrometry and stored at −20°C.

SCCmec typing of the MRSA isolates was performed under previously described conditions to identify the five main SCCmec structures, I-V [22], [23], and the SCCmec-IV subtypes were also identified [5], [24]. As reference strains, COL, N315, MW2, E-MRSA-16, JCSC2172, JC1C4788, WIS, and 85/2082 were included; these strains were supplied by NARSA (Network on Antimicrobial Resistance in *S. aureus*) and the National Institute of Health in Colombia.

### Multilocus Sequence Typing (MLST)

MLST was conducted using primers and protocols described previously [7]. Seven housekeeping gene fragments (402–516 bp) were amplified and sequenced. The sequence type (ST) was determined for each strain by comparing the sequence obtained to known alleles at each locus in the MLST database (http://saureus.mlst.net). The STs sharing 100% genetic identity in at least five of the seven MLST loci were grouped into a clonal complex (CC), and the ancestral founders were identified using the eBURST v3 program. To identify further relationships between the isolates, a minimal spanning network of MLST data was generated using TCS v2.1.

### Polymorphism of the X region encoding protein A (*spa* typing)

The X region of the *spa* gene was amplified and sequenced as described previously [25]. The sequences were analyzed with the Ridom StaphType software program (version 1.4; Ridom, GmbH, Wurzburg, Germany [http://spa.ridom.de/index.shtml]) to obtain the *spa* type for each strain.

### Accessory gene regulator (*agr*) typing

To distinguish between the *agr* groups I, II, III, and IV, the *agr* gene was amplified in each MRSA isolate using the primers and conditions described by Peacock *et. al.* [26]. In those reactions, the amplification of a 739-bp DNA fragment corresponded to *agr* group I, that of a 691-bp fragment corresponded to *agr* group II, that of a 712-bp fragment corresponded to *agr* group III, and that of a 683-bp fragment corresponded to *agr* group IV.

### Virulence gene profiles

The isolates were screened for the following 13 staphylococcal virulence genes: the staphylococcal enterotoxin genes (*seb, seq, sek*), the exfoliative toxin genes (*eta, etb*), the PVL genes (*lukF/S-*PV), the gamma-hemolysin gene (*hlg*), and the adhesin genes (*fnb-A, clf-A, clf-B, ica, cna*) as previously described [26], [27]. The DNA sequencing was performed to confirm the presence of *pvl* genes in a subset of 5 strains carrying the SCCmec type I.

### Ethics Statement

The research protocol and informed consent were approved by the Bioethics Committee at the Universidad Industrial de Santander in accordance with the ethical standards of the 1964 Declaration of Helsinki (Acta No. 15 27/08/2007). Each child's parents or guardians provided written informed consent for the review of medical information, and all the information was confidential.

## Results

Fifty-three MRSA isolates were obtained from patients 2 months to 13 years old with local or systemic infections, and all the isolates were positive for the *nuc* and *mecA* genes. Most of the MRSA isolates were susceptible to the majority of the antibiotics tested; only one isolate presented a multi resistant phenotype (resistant to 4 antibiotics); 47% of the MRSA isolates exhibited resistance to tetracycline, 38% to erythromycin, 26% to ciprofloxacin, 25% to clindamycin and 2% to TMP-SMX. All the strains were susceptible to vancomycin and rifampicin.

Clinical analysis was performed in 81% of the documented infections. Among the 43 infections in which the origin was documented, 46% were CA-MRSA, 35% were HAHO-MRSA, and 19% were HACO-MRSA.

### Molecular typing

Three SCCmec types were found among the isolates: type I in 15%, IVa in 4% and IVc in 75%. Three isolates were not typeable (NT) among the five SCCmec types searched for in the study (6%) (Table 1). The isolates classified as CA-MRSA and HACO-MRSA predominantly harbored the SCCmec IVc/a (80% and 88%, respectively); however, these SCCmec types were also carried in a high percentage of HAHO-MRSA isolates (74%). The SCCmec type I caused 20% of CA-MRSA cases and 20% of HAHO-MRSA cases.

**Table 1:** Clonal complexes and the relationship among the molecular types of MRSA isolates from pediatric patients.

Clonal complex (CC)	MLST^1^	*agr* group	SCC*mec* type	*spa* type	No.
**CC8**	ST8	I	IVc	t008	4
				t024	22
				t1705	1
				t2953	1
				t10796^2^	1
				t10799^3^	1
			IVa	t1635	1
		III	I	t024	2
		NT	IVc	t024	1
				NT	1
			NT		1
	ST254	I	IVc	t024	1
			I	t024	1
	ST552	I	IVc	t024	1
	ST931	I	I	t024	1
**CC5**	ST5	II	I	t0149	3
			IVc	t0149	3
		III	NT	t0149	1
			IVc	t0149	1
		NT	I	t4088	1
**CC78**	ST88	I	NT	t1814	1
		III	IVa	t1814	1
	ST188	I	IVc	t189	1
**CC80**	ST80	I	IVc	t044	1

1 MLST allelic profile in the order of *arcC*-*aroE*-*gypF*-*gmk*-*pta*-*tpi*-*yqiL*;

2 New *spa* type (allelic profile: 11-19-12-12-34-22-24-34-22-25);

3 New *spa* type (allelic profile: 11-12-156-17-34-24-34-22-25) after comparing the sequences and allelic profiles with those deposited in the spa typing website (http://spa.ridom.de/index.shtml).

**Figure 1:**
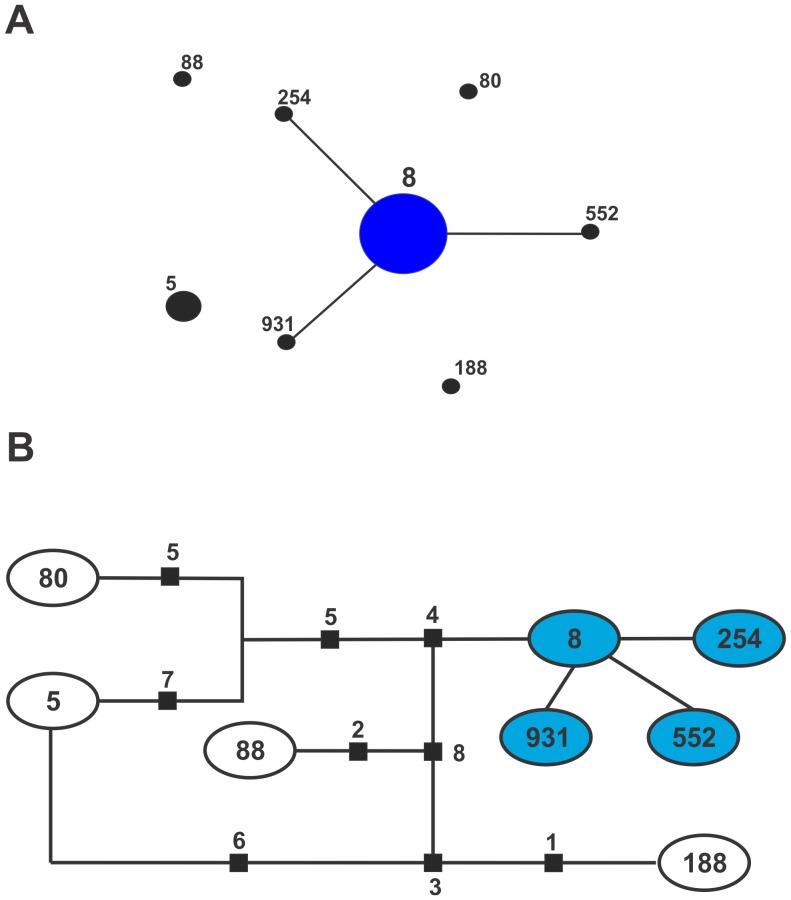
Distribution of STs in the clonal complexes. (**A**) The eBURST application of the MLST data from all of the isolates analyzed in this study. The numbers represent STs. STs that are linked by a line belong to the same cluster. Circle sizes are proportional to the number of strains within the ST. (**B**) Minimal spanning network of MLST data from the same isolates analyzed in (A). Circles represent STs, with the numbers inside the circles. Branches represent a single nucleotide change between neighboring STs. Black squares indicate multiple nucleotide changes between adjoining STs, with the number of differences indicated by the adjacent numbers. Similar-colored circles represent STs belonging to the same cluster, as in (A). Uncolored circles are singletons by eBURST analysis.

Using MLST, the 53 strains of this study were grouped in eight different STs; the majority (36 isolates) were ST8 (67.9%), and nine isolates were ST5 (17%) (Table 1). The MLST data analysis by eBURST v3 grouped the isolates into one CC and four singletons. The CC was conformed for ST8 as a probable founder genotype (ancestral genotype) and for ST254, ST552 and ST931 as single locus variants (SLV) from ST8 (Figure 1A). A minimal spanning network was generated to evaluate the nucleotide differences between the isolates. This methodology revealed that the strains clustered by eBURST differed by one polymorphism (Figure 1B), and the singletons differed by more than seven nucleotides.

The *spa* typing discriminated MRSA in twelve *spa* types, and two *spa* types were the most prevalent: t024 (56.6%) and t149 (15.1%). Two new repeat sequences were identified, and they were assigned numbers by the SpaServer (spaServer.ridom.de): t10786 and t10799.

Three *agr* groups were found to be the most frequent: *agr* group I (71.7%), followed by *agr* group II (11.3%) and *agr* group III (9.4%). Four isolates were not classified in any of the established *agr* groups.

The association between various molecular types was observed. The ST8 genotype was related primarily with *spa* t024 (25/36) and t008 (4/34) and less frequently with five *spa* types: t1635, t1705, t2953, t10796 and t10799. The ST5 genotype was associated primarily with *spa* type t149 (8/9) (Table 1).

Combining the SCCmec and MLST genotypes identified four clonal complexes (CC): CC8, CC5, CC80 and CC78 (Table 1). Seven different clones belonged to CC8 (75.5%); ST8-MRSA-IVc was the most frequent clone (60.4%). CC5 included ST5-MRSA-I (7.5%) and ST5-MRSA-IVc (7.5%). CC78 included ST88-MRSA-IVa (2%). Three isolates did not cluster into any of these four clone complexes because they were not typeable by SCCmec.

### Correlation between molecular types and virulence genes

**Figure 2:**
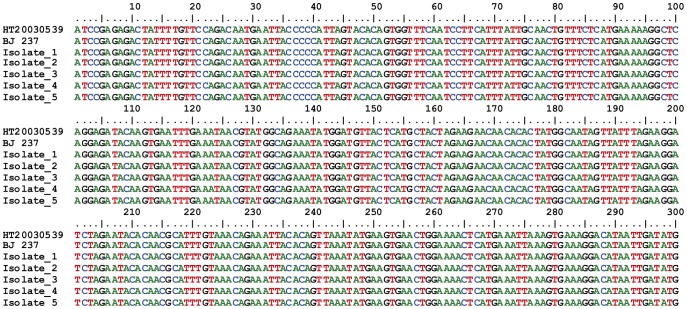
Alignment of the *pvl* sequences detected in a MRSA isolate harboring SCCmec type I.

All the isolates were carriers of at least one toxin gene. The most prevalent toxin genes detected were *hlg* (100%), *sek* (92%), *pvl* (88%), *seq* (49%) and *seb* (40%) (Table 2). The *tst-1* gene was not detected in any of the MRSA isolates. The adhesion genes were presented in most of the MRSA isolates; 89% carried the *clf-A* gene, 87% carried *clf-B*, 83% carried *fnb-A*, and 83% carried *ica* (Table 2). None of the MRSA isolates carried the *cna* gene. Some virulence genes were associated with specific molecular types (Table 2); *sek* and *pvl* were predominantly associated with SCCmec I and SCCmec IVc; *agr* group I; and ST8 and ST5. In the SCCmec I *pvl*+ strains, DNA sequencing confirmed the presence of the *pvl* gene (Figure 2). The *seq* gene was mainly carried by the isolates with SCCmec IVc, *agr* group I, and ST8. The *seb* gene was associated with SCCmec IVc, *agr* group II and ST5. A total of ten virulence gene profiles were observed. The most prevalent were *seq-sek-hlg* (22.6%), *sek-hlg* (22.6%), *seb-seq-sek-hlg* (18.9%) and *seb-sek-hlg* (15.1%) (Table 3).

**Table 2:** Virulence gene distribution among the molecular types of MRSA isolates from pediatric patients.

Virulence gene	MLST No. (%)								*agr* group No. (%)				SCC*mec* type No. (%)				No. (%)
	ST8	ST5	ST254	ST80	ST88	ST188	ST552	ST931	I	II	III	NT	I	IVa	IVc	NT	
***pvl***	34 (71)	7 (15)	2 (4)	1 (2)	2 (4)	–	1 (2)	1 (2)	35 (73)	5 (10)	4 (8)	4 (8)	7 (15)	2 (4)	37 (77)	2 (4)	48 (90)
***seb***	14 (67)	6 (29)	1 (5)	–	–	–	–	–	13 (62)	5 (24)	1 (5)	2 (10)	5 (24)	–	16 (76)	–	21 (40)
***seq***	23 (88)	1 (4)	2 (8)	–	–	–	–	–	23 (89)	1 (4)	–	2 (8)	2 (8)	1 (4)	22 (85)	1 (4)	26 (49)
***sek***	36 (73)	7 (14)	2 (4)	1(2)	–	1 (2)	1 (2)	1 (2)	37 (76)	4 (8)	4 (8)	4 (8)	7 (14)	1 (2)	39 (80)	2 (4)	49 (92)
***eta***	5 (56)	2 (22)	–	–	2 (22)	–	–	–	5 (56)	1 (11)	1 (11)	2 (22)	1 (11)	2 (22)	4 (44)	2 (22)	9 (17)
***etb***	–	–	1 (100)	–	–	–	–	–	1 (100)	–	–	–	1 (100)	–	–	–	1 (2)
***fnb-A***	29 (66)	8 (18)	2 (5)	1 (2)	2 (5)	1 (2)	1 (2)	–	31 (71)	5 (11)	5 (11)	3 (7)	6 (14)	1 (2)	34 (77)	3 (7)	44 (83)
***clf-A***	33 (70)	9 (19)	1 (2)	1 (2)	1 (2)	1 (2)	1 (2)	–	34 (72)	6 (13)	5 (11)	2 (4)	6 (13)	2 (4)	38 (81)	1 (2)	47 (89)
***clf-B***	33 (72)	6 (13)	2 (4)	–	2 (4)	1 (2)	1 (2)	1 (2)	36 (78)	3 (7)	4 (9)	3 (7)	6 (13)	2 (4)	35 (76)	3 (7)	46 (87)
***ica***	30 (68)	8 (18)	2 (5)	1 (2)	1 (2)	1 (2)	1 (2)	–	33 (75)	5 (11)	5 (11)	1 (2)	6 (14)	2 (5)	35 (80)	1 (2)	44 (83)

**Table 3:** Virulence gene profiles associated with different SCCmec types and *agr* groups of MRSA isolates from pediatric patients.

Virulence gene profiles	SCCmec type	*agr* group	No. (%)
*seq-sek-hlg*	IVc	I	11 (20.8)
*seq-sek-hlg*	IVc	NT	1 (1.9)
*seb-seq-sek-hlg*	IVc	I	9 (17)
*seb-seq-sek-hlg*	I	II	1 (1.9)
*sek-hlg*	IVc	I	7 (13)
*sek-hlg*	IVc	II	1 (1.9)
*sek-hlg*	IVc	III	1 (1.9)
*sek-hlg*	I	I	1 (1.9)
*sek-hlg*	I	III	1 (1.9)
*sek-hlg*	NT	III	1 (1.9)
*seb-sek-hlg*	IVc	I	5 (9.4)
*seb-sek-hlg*	IVc	NT	1 (1.9)
*seb-sek-hlg*	I	II	1 (1.9)
*seb-sek-hlg*	I	III	1 (1.9)
*seq-sek-eta-hlg*	IVc	I	2 (3.8)
*seq-sek-eta-hlg*	IVa	I	1 (1.9)
*seq-sek-eta-hlg*	NT	NT	1 (1.9)
*seb-sek-eta-hlg*	IVc	II	1 (1.9)
*seb-sek-eta-hlg*	I	NT	1 (1.9)
*seq-sek-etb-hlg*	I	I	1 (1.9)
*sek-eta-hlg*	IVc	I	1 (1.9)
*eta-hlg*	IVa	III	1 (1.9)
*eta-hlg*	NT	I	1 (1.9)
*seb-hlg*	IVc	II	1 (1.9)
**Total**			**53 (100)**

## Discussion

The genotypic characterization of MRSA has been widely used in epidemiological studies to understand the behavior and distribution of clones and CC and to establish and implement appropriate control measures to prevent the spread of MRSA infection. Given the strong effect of MRSA infections on pediatric patients, who have increased morbidity and mortality, it is necessary to genotype the isolates and to correlate their genotypes, phenotypes and clinical features.

In this study, ST8-MRSA-IVc was the predominant CC. In Latin America, the ST8-SCCmec-IVc clone is a variant of USA-300 referred to as USA300-LV, which has been detected previously in Colombia, Ecuador, Venezuela, Peru, Argentina and Trinidad and Tobago [17], [28], [29]. It is characterized by the presence of SCCmec IVc, the absence of ACME and frequent resistance to tetracycline [29]. Although SCCmec-IVc has been detected mainly in CA-MRSA clones, most of the MRSA isolates in our hospital were identified as ST8-MRSA-IVc, and only a small percentage of isolates carried the SCCmec type I. These findings confirm the establishment and high prevalence of CA-MRSA strains, particularly the USA300-LV clone, in hospitals, as previously reported [17], [28].

Although the Pediatric clone has been characterized as presenting a sensitivity profile, the ST5-MRSA-IVc/a clones described in this work presented a resistance profile against non-beta lactam antibiotics, in agreement with previous reports in Colombia [16]. The isolates of the ST5-MRSA-I clone had a profile of multidrug resistance and were associated with nosocomial infections, similar to isolates described previously in Colombia and in other Latin American countries such as Chile, Argentina and Paraguay [30], [31], [32], [33], [34], [35]. Although in lower percentages, the ST931-MRSA-I, ST552-MRSA-IVc, and ST80-MRSA-IVc clones were detected and are reported herein for the first time in Colombia, demonstrating the circulation of different clones in our hospital.

Based on their *agr* polymorphisms, the isolates were classified into four groups, I-IV. The *agr* group I was identified in the majority of the analyzed isolates, as previously reported in other countries, with a predominance of this group among the clinical isolates [36], [37], [38]. Other studies have reported either *agr* group III as being predominant among CA-MRSA isolates [39] or an equitable distribution of the four *agr* groups [40], [41]. The polymorphisms in the *agr* or altered expression of the *agr* system have been linked to the phenotype of intermediate resistance to vancomycin (VISA) and heterogeneous resistance (hetero-VISA) [40], [42]. In our study, all of the MRSA isolates were susceptible to vancomycin.

Staphylococcal protein A (SpA) is anchored in the cell wall and is a virulence factor present in most *S. aureus* isolates [43]. The polymorphism found in region X of the gene *spa* has been used in the characterization of *S. aureus*, showing a discriminatory power comparable to other methods, such as PFGE and MLST [44]. A total of twelve *spa* types were found in this study, and two of them were not previously reported (t10796 and t10799). The *spa* t024 was more frequently associated with ST8, ST254, ST552 and ST931 (CC8), as described previously [45]. In Colombia, a previous study identified a higher frequency of t1610 and the absence of t024 [17]. The *spa* type t149 is one of the most common types in South America and has been related with the Cordobes/Chilean clone [34], [46]. In our study, t149 was the second most common type and was associated with ST5, which was similar to the results of a previous study on HA-MRSA [34]. Other *spa* types identified include t189 and t1635; the latter was associated with ST8. Although Jimenez *et al.* also reported these *spa* types in Colombia, the types were related with ST188 and ST923 [17].

The Panton-Valentine leukocidin (PVL) has been associated with the progression to severe infections such as necrotizing pneumonia, bone and joint infections and bacteremia [27], [47], [48], [49]. A total of 75% of our isolates, most commonly the SCCmec IVc isolates, were *pvl* +; some of the SCCmec I and IVa isolates were also *pvl* +. These results are in agreement with some reports in the United States, China and Lebanon, where most MRSA carriers of SCCmec IVa and IVc are *pvl*+ [50], [51], [52]. The detection of *pvl* genes in SCCmec I strains is not common, and these genes have not been detected in HA-MRSA strains (SCCmec I, II or III) [53], [54]. In Colombia, SCCmec I *pvl*+ isolates have been reported [55], although in a lower percentage than in our hospital.

*S. aureus* produces a large number of toxins including hemolysins, staphylococcal enterotoxins (SEs), exfoliative toxins (ETA and ETB) and the toxin of toxic shock syndrome-1 (TSST-1), which causes food poisoning, enterocolitis, scalded skin syndrome and toxic shock, among other conditions [56]. The majority of MRSA isolates were found to harbor the *hlg, sek, seb* and *seq* genes, but no association was found between SCCmec type and SEs genes, as described previously [38], [57]. The low frequency of *eta* and *etb* coincides with the results of a study that found these genes in 5% of *S. aureus* [58]. Some isolates carried multiple toxins, and a total of ten toxin gene profiles were found in our study. The most prevalent profiles were *seq-sek-hlg*, *sek-hlg*, *seb-seq-sek-hlg* and *seb-sek-hlg*. There was a great variety of toxin combinations, but contrary to previous reports [38], [57], [59], no statistical association was found between toxin gene profiles with a molecular type or SCCmec type.

*S. aureus* expresses many surface proteins of the MSCRAMM (microbial surface components recognizing adhesive matrix molecules) family, which specifically recognize and bind to the extracellular matrix components of the host. Among the five adhesion molecules evaluated in this work, *clf*-A, *clf*-B and *fnb*-A were the most prevalent. The *clf-A* and *clf-B* genes encode an adhesin protein for fibrinogen and *fnb*-A for fibrinogen and fibronectin. The high percentage observed in our study is consistent with the fact that these genes are ubiquitously carried by different *S. aureus* lineages [26] and that these genes have been reported to play a determinant role in bacterial virulence [60]. The *cna* gene was not detected in any of our isolates, and although its role in the pathogenesis of bone infections is well documented, it appears to play a less determinant role in skeletal muscle infection [61].

SCCmec IVc was predominant in the isolates classified as CA-MRSA, HACO-MRSA and HAHO-MRSA. This finding confirms a high prevalence of SCCmec IVc isolates (characteristic of CA-MRSA) in Colombian hospitals and their circulation as nosocomial pathogens causing HACO-MRSA and HAHO-MRSA. Although all of the isolates were from hospitalized patients, SCCmec I was detected in a low percentage of isolates (15%) and was associated with all three infection types. All of the pediatric patients developed moderate or severe infections, predominantly skin and soft tissue infections. In some cases, the patients presented with invasive disease such as bacteremia, and in all cases, the patients had to be treated and hospitalized in an advanced level hospital. The severity of the MRSA infections observed in the pediatric patients included in the analysis might be related to the high frequency of virulence factors carried by the isolates. In previous studies, a number of genes such as *pvl, hlg, fnbA, cna, sdrE, hlg, sej, eta*, and *ica* have been associated with the development of invasive disease, contributing independently to the virulence of *S. aureus* [26], [62].

We identified the presence of four CC among the MRSA isolates from pediatric patients in a hospital in Colombia. The molecular type ST8-MRSA-IVc-*pvl* + was the most prevalent. New *spa* types were detected for the first time in Colombia, as well as two new repeat sequences: *spa* t10799 and t10796. Different toxin profiles were identified that showed great diversity among the isolates, and the majority of them carried one or more toxin genes. MRSA carried numerous adhesion molecules with other virulence factors such as toxins that are essential for the establishment of clinical disease with deep tissue involvement and severe progression, such as those observed in the pediatric patients of this study. We have confirmed the presence of CA-MRSA type strains (ST8-MRSA-IVc-*pvl*+) associated with pediatric infections in our hospital.
